# Multiscale Metal Oxide Particles to Enhance Photocatalytic Antimicrobial Activity against *Escherichia coli* and M13 Bacteriophage under Dual Ultraviolet Irradiation

**DOI:** 10.3390/pharmaceutics13020222

**Published:** 2021-02-06

**Authors:** Su-Eon Jin, Hyo-Eon Jin

**Affiliations:** 1 Research Institute for Medical Sciences, College of Medicine, Inha University, Incheon 22212, Korea; 2 College of Pharmacy, Ajou University, Suwon 16499, Korea

**Keywords:** multiscale metal oxide particles, dual UV, photocatalytic antimicrobials, *E. coli*, M13 bacteriophage

## Abstract

Antimicrobial activity of multiscale metal oxide (MO) particles against *Escherichia coli* (*E. coli*) and M13 bacteriophage (phage) was investigated under dual ultraviolet (UV) irradiation. Zinc oxide (ZnO), magnesium oxide (MgO), cuprous oxide (Cu_2_O), and cupric oxide (CuO) were selected as photocatalytic antimicrobials in MO particles. Physicochemical properties including morphology, particle size/particle size distribution, atomic composition, crystallinity, and porosity were evaluated. Under UV-A and UV-C irradiation with differential UV-C intensities, the antimicrobial activity of MO particles was monitored in *E. coli* and phage. MO particles had nano-, micro- and nano- to microscale sizes with irregular shapes, composed of atoms as ratios of chemical formulae and presented crystallinity as pure materials. They had wide-range specific surface area levels of 0.40–46.34 m^2^/g. MO particles themselves showed antibacterial activity against *E. coli*, which was the highest among the ZnO particles. However, no viral inactivation by MO particles occurred in phage. Under dual UV irradiation, multiscale ZnO and CuO particles had superior antimicrobial activities against *E. coli* and phage, as mixtures of nano- and microparticles for enhanced photocatalytic antimicrobials. The results showed that the dual UV-multiscale MO particle hybrids exhibit enhanced antibiotic potentials. It can also be applied as a next-generation antibiotic tool in industrial and clinical fields.

## 1. Introduction

Metal oxide (MO) particles have been highlighted as nano-antibiotics against pathogenic microorganisms for enhanced disinfection [1,2]. They exhibit wide-spectrum antimicrobial activities against bacteria as well as viruses, and even against antibiotic-resistant microorganisms [3,4]. The antimicrobial functions of MO particles are displayed by their adsorption potentials to biomembranes of microorganisms, owing to their surface properties [5,6]. Under ultraviolet (UV) irradiation, reactive oxygen species (ROS) produced by MO particles induce germicidal toxicity of nucleic acid breaks in microorganisms. In MO particles, biological and toxicological responses against microorganisms are primarily mediated by physicochemical characteristics under non-UV or UV irradiation. Specifically, their physicochemical characteristics including morphology, particle size, atomic composition, crystallinity, and porosity are the major factors to affect the responses of microorganisms inducing antimicrobial actions [7]. Among the properties of MO particles, their photocatalytic antimicrobial activity of MO particles can be mainly affected by particle size variation [8,9]. Therefore, the antimicrobial activity of multiscale MO particles ranging from nano- to microsized levels should be screened.

Particle size variety has been studied in photocatalytic antimicrobial activity of MO particles for enhanced disinfection [7,10]. In the case of MO nanoparticles (NPs, <100 nm) including zinc oxide (ZnO), zinc titanate (ZnTiO_3_), magnesium oxide (MgO), and cupric oxide (CuO) used as photocatalysts, they showed enhanced antimicrobial activity and blocked bacterial regrowth as compared to UV irradiation alone [11,12]. On the contrary, low antimicrobial activities were detected in MO microparticles (MPs, >100 nm) against *Escherichia coli* and *Staphylococcus aureus*, which ranged from 0.1 to 0.8 μm in diameter with specific surface area of 0.85–26.0 m^2^/g [9]. However, the photocatalytic disinfection potential of MO MPs can be improved in ROS generation under UV irradiation based on the porosity via multiple scattering phenomena for enhanced mass transfer and exchange rate [13,14]. In addition to MO NPs or MPs alone, photocatalytic antimicrobial activity of multiscale MO particles can be monitored as mixtures of MO NPs and MPs.

The hybridization of MO particles to dual UV, UV-A (315–400 nm) and UV-C (100–280 nm), is one of the most ecofriendly disinfection techniques, which facilitates wide-spectrum antimicrobial activity based on combined wavelengths [15,16]. Although UV-A is used for the removal of harmful insects and worms after a long exposure [17], UV-C is a powerful germicidal wavelength range, specifically 253 nm, for pathogenic microorganism disinfection [18]. Enhanced antibiotic potential of dual UV is described by a combination of MO NPs as photocatalysts, compared to single UV or antibacterial agent alone [19,20]. A dual UV and photocatalyst hybrid system can prevent regrowth or reactivation of bacteria and viruses after single UV irradiation, killing antibiotic-resistant microorganisms.

In the present study, we investigated whether multiscale MO particles, a mixture of MO NPs and MPs could have an enhanced antimicrobial activity coupled with non-UV or UV irradiations. ZnO, MgO, Cu_2_O, and CuO particles were selected to screen antimicrobial activity. Physicochemical characteristics of multiscale MO particles were evaluated in terms of morphology, particle size/particle size distribution, atomic composition, crystallinity, and porosity. Antimicrobial activity test was performed in *E. coli* and M13 bacteriophage (phage), which were used as model microorganisms. The mechanisms underlying antimicrobial action were further discussed.

## 2. Materials and Methods

### 2.1. Chemical Reagents

MO particles consisting of ZnO [ZnO-(1) (powder, <5 μm, 99.9%) and ZnO-(2) (nanopowder, <100 nm)], MgO [MgO-(1) (calcined, ≥97.0%) and MgO-(2) (fused, 150–325 mesh, ≥95%)], Cu_2_O (powder, ≤7 μm, 97%), and CuO (powder, <10 μm, 98%) were purchased from Sigma-Aldrich (St. Louis, MO, USA). Absolute ethanol (99.9%) and isopropyl alcohol were obtained from Sigma-Aldrich (USA). Luria-Bertani (LB) medium and agar were obtained from BD Biosciences (Franklin Lakes, NJ, USA). All chemicals were of reagent grade and used without further purification. Deionized water was obtained using a water purification system (Milli-Q, Millipore, Billerica, MA, USA).

### 2.2. Field Emission-Scanning Electron Microscopy (FE-SEM) with Energy-Dispersive X-ray Spectroscopy (EDS)

Morphologies of MO particles were monitored using a FE-SEM (JSM-7100F, Jeol Ltd., Tokyo, Japan). It was operated at an acceleration voltage of 15.0 kV. Magnifications were set at 5000–50,000. Samples were investigated after platinum coating. EDS analysis was also performed at three points on the surface of MO particles to quantitatively determine the compositions of elements.

### 2.3. Powder X-ray Diffractometry (PXRD)

The crystallinities of MO particles were analyzed using a PXRD. The PXRD patterns of the particles were recorded from 20 to 80 2θ (degree, °) using a high resolution X-ray diffractometer (HR XRD, SmartLab, Rigaku, Tokyo, Japan) with CuΚα radiation. The data were collected and analyzed in SmartLab Studio (Rigaku).

### 2.4. Brunauer–Emmett–Teller (BET) Analysis

Mesopores of MO particles were analyzed from nitrogen adsorption-desorption isotherms using a Qudrasorb SI (Quantachrome Instruments, Boynton Beach, FL, USA). Each sample was weighed at 1.0–3.0 g. Experimental results of specific surface area, total pore volume, and average pore diameter were calculated using ASiQwin software (Quantachrome Instruments) based on the BET theory.

### 2.5. Dual UV Irradiation in Collimated Beam Device (CBD)

CBD was prepared using a dual UV lamp of UV-A and UV-C (ECOSET Co., Ltd., Ansan, Korea), controlling UV-C intensity by lamp surface coating [11]. The electronic controller for 40 W/m^2^ was connected to CBD. Fans were attached at both ends to minimize the heat generation of the UV lamp. UV dose (J/m^2^) was calculated from the intensity (W/m^2^) of the UV lamp and exposure time (s) after obtaining UV intensity using a spectrometer (Jaz System, Ocean Optics, Inc., Orlando, FL, USA) with software (Spectra Suite, Ocean Optics, Inc.).

### 2.6. Antimicrobial Activity Test in E. coli and Phage

Antimicrobial activities of the MO particles and dual UV-MO particle hybrids were investigated in *E. coli* and phage as the model microorganisms of bacteria and viruses, respectively. MO particles or dual UV alone were used as controls. Dispersed MO particles in water (1 mg/mL) were added to *E. coli* (10^4^ colony forming unit, CFU). After incubation in the dark for 30 min, the samples (1 mL) were collected, added to LB/agar medium, and poured into plates. The resultant plate samples were incubated in the dark at 37 °C overnight. In the case of phage, MO particles in water (1 mg/mL) were mixed with phage (10^4^ plaque forming units, PFU) and incubated for 30 min. Then, 100 µL of the samples were collected and incubated with overnight cultured bacteria at room temperature for 60 min. After mixing the top agars with those, they were poured onto LB/isopropyl β-d-1-thiogalactopyranoside (IPTG)/5-bromo-4-chloro-3-indolyl-β-D-galactopyranoside (X-gal) plates and incubated at 37 °C overnight. For photocatalytic antimicrobial activity, MO particles were irradiated under dual UV for 10 or 30 s while they were incubated with *E. coli* or phage for 30 min. After incubation, each sample for *E. coli* or phage was collected and processed as mentioned above. The colonies and phage plaques were counted using Image J (NIH) after their images were obtained.

### 2.7. Statistical Analysis

The results are expressed as the means ± standard deviation. The statistical differences among the groups were tested using Student’s *t*-test. A *p*-value less than 0.05 was considered to be statistically significant.

## 3. Results

### 3.1. Morphology and Particle Size Distribution

**Figure 1:**
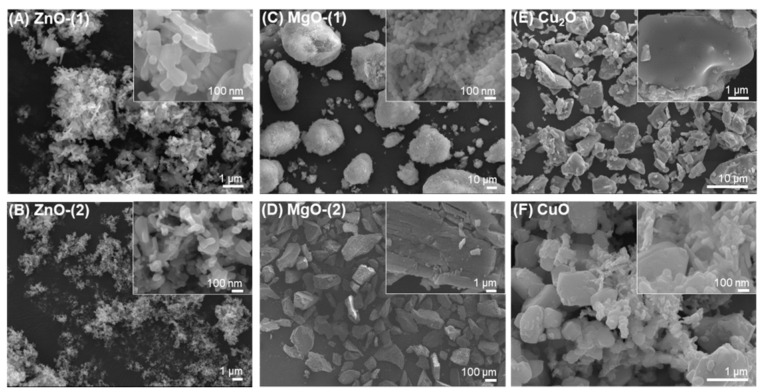
FE-SEM images of metal oxide (MO) particles: (**A**) ZnO-(1), (**B**) ZnO-(2), (**C**) MgO-(1), (**D**) MgO-(2), (**E**) Cu_2_O, and (**F**) CuO.

MO particles of ZnO, MgO, Cu_2_O, and CuO formed various irregular shapes such as needles, rods, and spheres to develop additive particle clusters with interparticular pores (Figure 1). ZnO-(1) (Figure 1A) and ZnO-(2) (Figure 1B) particles had shapes similar to needles or rods. MgO-(1) particles (Figure 1C) had nanoscale-sized shapes of spheres or cubes, which generated submicron-sized spherical clusters. However, MgO-(2) particles (Figure 1D) showed cuboidal crystalline shape. Cu_2_O (Figure 1E) and CuO (Figure 1F) particles were mixtures of irregular shaped NPs and had curved or angular particles of submicron size.

In particle size distribution, MO particles had nano- to microscale sizes of 84.6 nm–0.706 μm for ZnO-(1) and 76.9–153.8 nm for ZnO-(2) particles [ZnO-(1) > ZnO-(2)]; 50–76.9 nm for MgO-(1) and 8.0–138 μm for MgO-(2) particles [MgO-(1) < MgO-(2)]; 1.2–6.0 μm for Cu_2_O and 50.0 nm–0.577 μm for CuO particles (Cu_2_O ≥ CuO). Specifically, ZnO-(1) and CuO particles were multiscale mixtures of NPs and MPs. ZnO-(2) and MgO-(1) particles were NPs, and MgO-(2) and Cu_2_O particles were MPs. However, in MgO particles, MgO-(1) particles generated particle clusters ranged from 28 to 54 μm, which were smaller than MgO-(2) particles (>100 μm). In a hydrodynamic environment, MO particles generated large aggregates (<100 nm, >100 μm) in a number-weighted distribution mode (Figure S1). In ZnO and MgO particles, each particle size of aggregate was conversely displayed, compared to individual particle size, due to the differential particle aggregate formation capacity [ZnO-(1) < ZnO-(2); MgO-(1) > MgO-(2)] (Table S1). In the case of Cu_2_O and CuO particles, their aggregate sizes were similar to each other (Cu_2_O ≈ CuO).

### 3.2. Atomic Compositions

In EDS spectra (Figure S2), atomic compositions of MO particles were matched for pure chemical formula (Table 1). ZnO particles included 78.5–81.9% of Zn and 18.1–21.4% of O. MgO particles contained 61.1–63.5% of Mg and 36.5–38.9% of O. Cu_2_O and CuO particles had 88.7% of Cu and 11.3% of O for Cu_2_O, and 75.9% of Cu and 24.1% of O for CuO. Atomic compositions were similar in all the MO particles, irrespective of morphology and particle size. Although ZnO-(1) particles contained an Al contaminant of less than 0.05% in EDS, no Al contaminants were detected in XPS. Binding energy of ZnO-(1) particles were detected at 1022 and 1045 eV for Zn2p and 531 eV for O1s (Figure S3).

### 3.3. Crystallinity

**Figure 2:**
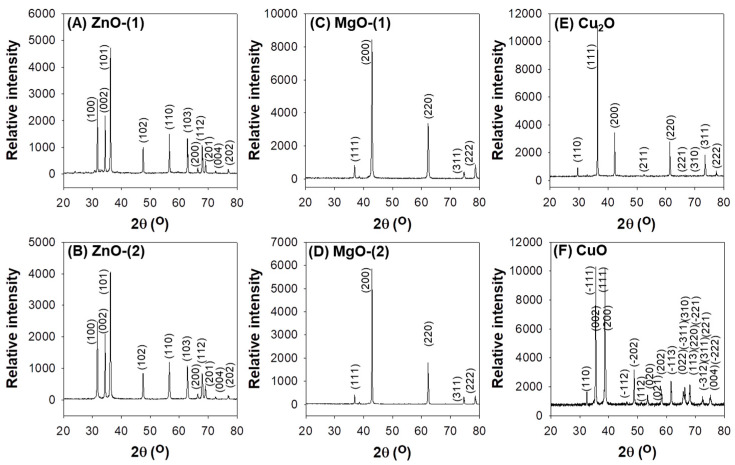
PXRD patterns of metal oxide (MO) particles: (**A**) ZnO-(1), (**B**) ZnO-(2), (**C**) MgO-(1), (**D**) MgO-(2), (**E**) Cu_2_O, and (**F**) CuO.

PXRD patterns described the high crystallinity of MO particles suggesting that they were pure materials with negligible impurities (Figure 2). MO particles showed hexagonal wurtzite structures for ZnO-(1) and ZnO-(2) particles (ICDD 01-080-0075, Figure 2A,B), cubic lattices for MgO-(1) (ICDD 01-071-3631, Figure 2C) and MgO-(2) (ICDD 00-045-0946, Figure 2D) particles, cubic polycrystalline for Cu_2_O particles (ICDD 01-071-3645, Figure 2E), and monoclinic phase for CuO particles (ICDD 01-089-5898, Figure 2F).

### 3.4. Porosity

Surface area, pore volume, and pore size of MO particles are listed in Table 2. Surface area levels were the highest in ZnO-(1) particles (46.34 m^2^/g), and the lowest in MgO-(2) particles (0.3997 m^2^/g) in order of ZnO-(1), MgO-(1), ZnO-(2), CuO, Cu_2_O, and MgO-(2) particles. In pore volume levels, MgO-(1) particles showed the highest (0.2883 cc/g) and MgO-(2) particles had the lowest (0.003593 cc/g) in order of MgO-(1), ZnO-(1), ZnO-(2), CuO, Cu_2_O, and MgO-(2) particles. Mesopore size levels of MO particles ranged from 9.540 to 45.76 nm in order of MgO-(1) (45.76 nm), MgO-(2) (35.96 nm), ZnO-(2) (29.86 nm), Cu_2_O (25.80 nm), CuO (21.02 nm), and ZnO-(1) (9.540 nm) particles.

### 3.5. Antimicrobial Activity of MO Particles

#### 3.5.1. Antibacterial Activity against *E. coli*

**Figure 3:**
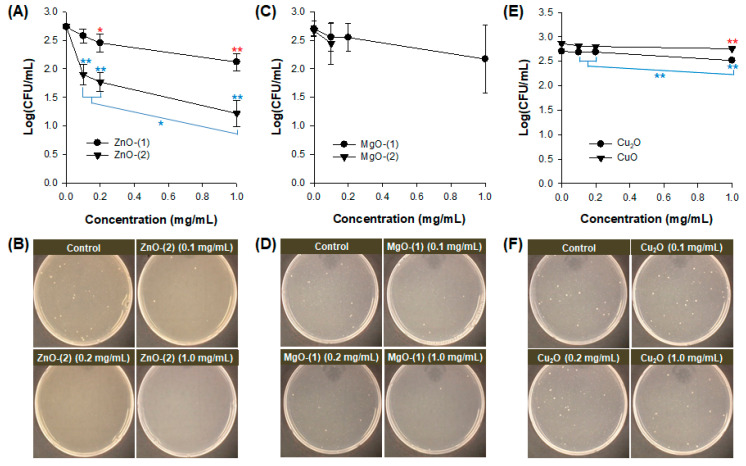
Antimicrobial effect of metal oxide (MO) particles against *Escherichia coli*: plots of concentration (mg/mL) versus log(CFU/mL) and representative plate images of (**A**,**B**) ZnO-(1), and ZnO-(2); (**C**,**D**) MgO-(1), and MgO-(2); (**E**,**F**) Cu_2_O, and CuO. *, *p* < 0.05; **, *p* < 0.01.

Antimicrobial effect of MO particles against *E. coli* was investigated without UV irradiation (Figure 3). In ZnO particles, both ZnO-(1) and ZnO-(2) particles showed a dose-dependent antibacterial activity against *E. coli* at 0.1–1.0 mg/mL (Figure 3A). Figure 3B displays the representative plate images after antibacterial activity test of ZnO-(2) particles against *E. coli*. Although MgO (Figure 3C,D), Cu_2_O and CuO (Figure 3E,F) particles also had significant antibacterial activity, they had lower growth inhibition levels against *E. coli* than those of ZnO-(2) particles. Antibacterial activity against *E. coli* was affected by physicochemical characteristics of MO particles. In ZnO particles, ZnO-(2) particles had enhanced antibacterial activity compared with ZnO-(1) particles depending on particle size [ZnO-(1) > ZnO-(2)]. In addition to particle size effect, antibacterial activity of MO particles was shown in order of ZnO > MgO > Cu_2_O = CuO particles based on the variety of all particles.

#### 3.5.2. Inactivation Activity against Phage

**Figure 4:**
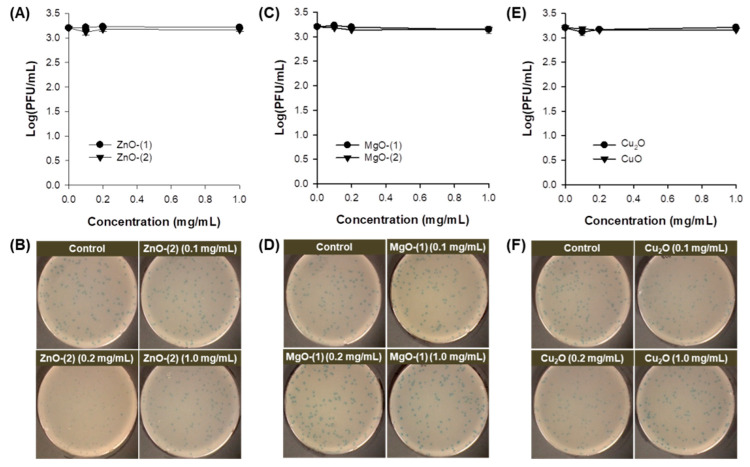
Antimicrobial effect of metal oxide (MO) particles against phage: plots of concentration (mg/mL) versus log(PFU/mL) and the representative plate images of (**A**,**B**) ZnO-(1), and ZnO-(2); (**C**,**D**) MgO-(1), and MgO-(2); and (**E**,**F**) Cu_2_O, and CuO.

Viral inactivation activity of MO particles was determined against phage in the dark (Figure 4). All particles of ZnO (Figure 4A,B), MgO (Figure 4C,D), Cu_2_O and CuO (Figure 4E,F) at 0.1–1.0 mg/mL showed no inactivation activity against phage after 30-min incubation. While virus plaques were inspected on plates as blue dots, no viral inactivation was confirmed in the representative plate images of ZnO-(2) particles (Figure 4B) along with all the other particles (Figure 4D,F) despite the enhanced antibacterial activity.

### 3.6. Dual UV-MO Particle Hybrid-Based Antimicrobial Activity

#### 3.6.1. Antibacterial Activity against *E. coli* under Dual UV Irradiation

**Figure 5:**
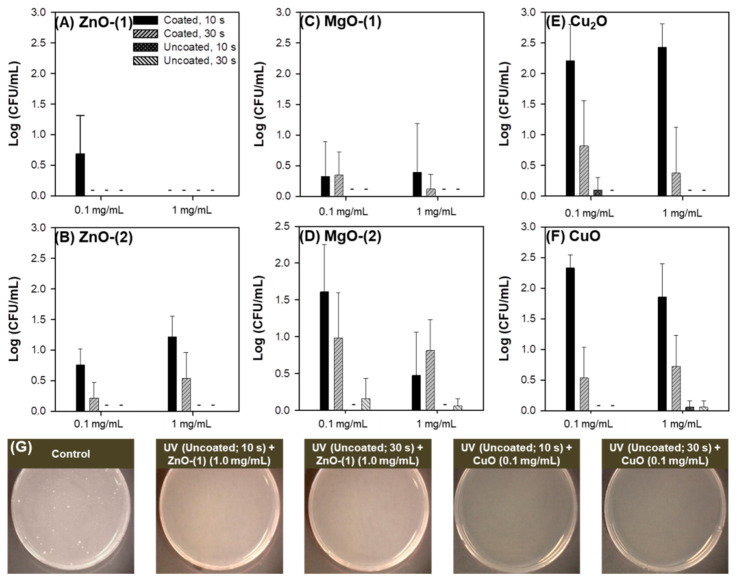
Antimicrobial effect of dual ultraviolet (UV)-metal oxide (MO) particle hybrids against *E. coli*: (**A**) ZnO-(1), (**B**) ZnO-(2), (**C**) MgO-(1), (**D**) MgO-(2), (**E**) Cu_2_O, and (**F**) CuO; (**G**) representative plate images of colonies. -, not detected.

Combination of dual UV and MO particles showed enhanced antibacterial efficacy against *E. coli* compared to MO particles alone (Figure 5). MO particles were used at 0.1 and 1.0 mg/mL. In dual UV irradiation for 10 and 30 s, coated or uncoated areas of UV lamp were applied as controllable factors for high or low UV-C intensity. Enhanced antibacterial inhibition against *E. coli* were displayed at higher concentration of MO particles (except ZnO-(2) and CuO), higher UV-C intensity, and longer UV exposure time. In MO particles, antibacterial activity under dual UV irradiation was the highest in ZnO-(1) particles and the lowest in MgO-(2) particles (ZnO-(1) > MgO-(1) > ZnO-(2) > CuO > Cu_2_O > MgO-(2)). Although ZnO-(1) particles were larger than ZnO-(2) particles, ZnO-(1) particles had superior antibacterial activity against *E. coli* than ZnO-(2) particles under dual UV irradiation. These results are contrary to those of antibacterial test without UV irradiation. It suggested that multiscale MO particles can enhance antibacterial activity as photocatalysts for ROS generation under dual UV irradiation.

#### 3.6.2. Inactivation Activity against Phage under Dual UV Irradiation

**Figure 6:**
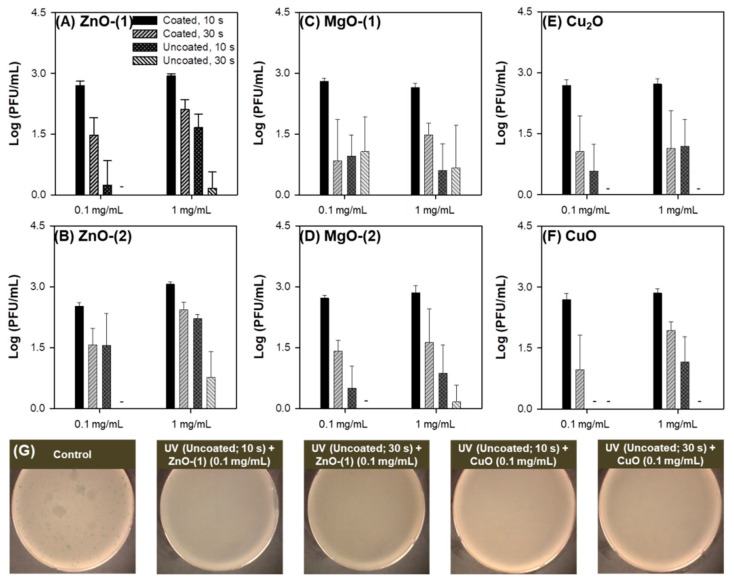
Antimicrobial effect of dual ultraviolet (UV)-metal oxide (MO) particle hybrids against phage: (**A**) ZnO-(1), (**B**) ZnO-(2), (**C**) MgO-(1), (**D**) MgO-(2), (**E**) Cu_2_O, and (**F**) CuO; (**G**) representative plate images of phage plaques. -, not detected.

Antimicrobial effect of dual UV-MO particle hybrids on phage was also investigated (Figure 6). Under dual UV irradiation, MO particles showed viral inactivation activity against phage. ZnO-(1), ZnO-(2), MgO-(2), Cu_2_O and CuO particles had viral inactivation activity even at lower concentration (0.1 mg/mL) for 30-s dual UV irradiation using lamp at uncoated side, except for MgO-(1) particles. Specifically, antimicrobial activity of CuO and ZnO-(1) particles in multiscale MO particles was enhanced under dual UV irradiation from uncoated area of UV lamp due to the high UV-C intensity based on combination effect (Figure 6G). MO particles at low concentration (0.1 mg/mL) were more efficient than those at high concentration (1.0 mg/mL) except MgO-(1) particles. Viral inactivation potential was the highest in CuO particles and the lowest in MgO-(1) particles among MO particles [CuO > Cu_2_O > ZnO-(1) = MgO-(2) > ZnO-(2) > MgO-(1)] under dual UV irradiation. Although MO particles themselves had antibacterial activity in a dose-dependent manner, UV irradiation platforms along with MO particles were necessary to inactivate viruses effectively.

## 4. Discussion

Antibiotic properties of MO particles have been mainly affected by physicochemical characteristics including morphology and particle size [1,21]. MO NPs are generally described as nano-antibiotics to combat multidrug resistance in pathogenic microorganisms [22], where antimicrobial activity is superior than that of MO MPs in most reports of antimicrobial activity tests. However, in some cases, MO MPs also showed strong antimicrobial activity based on their structural properties or combinations with other materials such as polymers and metallic compounds [23,24]. In the present study, antimicrobial activity of multiscale MO particles against *E. coli* and phage was investigated with or without dual UV irradiation to clarify the interactions between physicochemical characteristics, specifically particle size distribution, and biological responses in microorganisms for enhanced performance. Particle size distribution of MO particles in a multiscale mixture of NPs and MPs at nano-to-microscale size range (ZnO-(1) and CuO particles) was explored as a critical factor for their antimicrobial actions under dual UV irradiation.

First of all, morphology of MO particles affected antimicrobial activity via the interactions of the particles with microorganisms under dual UV irradiation [24]. MO particles showed needles or rods (Figure 1A,B), spheres or cubes (Figure 1C,D), and curved or angular shapes (Figure 1E,F). Compared to spherical NPs, MO NPs with high aspect ratios can be predicted as highly toxic materials with exposure risks for enhanced antimicrobial activity against bacteria and viruses [25,26]. However, irregular-shaped NPs of high aspect ratios (disc, rod, and needle) at 18.59–22.20 nm showed more cytotoxicity in MG-63 cells at 0.5 mg/mL than the spherical NPs after 24 h of incubation under the same conditions as those for antimicrobial activity enhancement [27].

Predicting antimicrobial effect of MO NPs against pathogenic microorganisms, MO NPs with smaller size (<100 nm) significantly offer higher antibiotic potentials, despite the association with morphology [28]. However, regarding aggregate generation of MO NPs in the hydrodynamic environment (Figure S1), multiscale MO particles can mimic nano- or microstructured aggregate generation containing nano-to-microscale particles (Table S1) and possibly induce multiple scattering for enhanced mass transfer and exchange rate under UV and visible light irradiation [29,30]. In photocatalysis for enhanced ROS generation, to induce oxidative stress against microorganisms, multiple scattering phenomena of multiscale MO particles increase photon utilization efficiency to improve an expected photo-redox performance.

Chemical composition and purity of MO particles also provide fundamental information on their antibiotic potential against pathogenic microorganisms [31]. They have a high priority even at macroscale particles in the physicochemical characteristics affected to biological responses, recommended by Organization for Economic Co-operation and Development (OECD) [21]. MO particles had atomic compositions as pure chemical formulae (Table 1), which were determined using EDS spectra (Figure S2). Specifically, binding energy levels of ZnO-(1) particles for investigating chemical states of surface defects were confirmed in XPS spectrum based on elemental surface composition suggesting the environmental interaction of particles influenced by air and carbon taping (Figure S3).

Crystallinity is one of key physicochemical characteristics to influence photocatalytic performance under UV and visible light irradiation based on band gap energy [32]. Biological interactions of MO particles as photocatalysts can be predicted by band gap energy that ranged from −4.12 to −4.84 eV of cellular redox potential, suggesting electron transfer between particle surfaces and cellular redox couples for oxidative stress [33]. MO particles of ZnO, MgO, Cu_2_O, and CuO had highly crystalline structures as pure materials of hexagonal wurtzite crystals, cubic lattices, cubic polycrystalline, and monoclinic phased crystals, respectively (Figure 2). Connecting to biological responses, crystal structures of MO particles can also provide antimicrobial potentials against pathogenic microorganisms.

In surface characteristics, porosity is essential to investigate the adsorption potential of MO particles in antimicrobial actions [34]. Large mesopores (10–50 nm) in MO particles induce fast biochemical adsorption on the particle surfaces, which has contributed to enhanced reaction rate, compared to small mesopores (2–10 nm). Depending on MO particle types, pore size strongly affected antimicrobial activity against microorganisms (Table 2).

For antimicrobial activity test, *E. coli* and phage have been extensively used as model microorganisms of bacteria and viruses [35,36]. *E. coli* is one of the most rapidly growing bacteria to easily quantify the colonies via UV-visible scanning for turbidity measurement and colony counting on agar plates [37,38]. It is a representative screening system for antibiotic materials in terms of metabolic process interruption leading to cell death or cell stasis. Expressing pck, acs, and atpAGD in ATP-consuming cycles of *E. coli*, cells are genetically sensitized to exogenous oxidative stress for bactericidal action. In addition, translation inhibition in *E. coli* induces bacteriostatic condition of decreasing cell respiration, which is similar to genetic cytochrome oxidase disruption in metabolic processes. Next, phage is a strong filamentous bacterial virus for antimicrobial activity test, which can induce multidrug resistance as a genetic reservoir [39]. However, they were recently studied to develop antimicrobial agents based on bioengineered phage-based bacterial infection combating multidrug resistance in bacteria [40]. Phage is also useful to quantify the plaque as an experimental system after bacterial infection. Therefore, *E. coli* and phage can be appropriate models for antibiotic screening and mechanistic study on how MO particles inhibit the bacterial and viral growth.

MO particles themselves have showed antimicrobial activity against microorganisms [22,35]. In the present study, spherical or cuboidal MgO particles presented lower antimicrobial activity against *E. coli* than ZnO, Cu_2_O, and CuO particles (Figure 3). Comparing with previous reports of MO NPs, ZnO NPs had 0.1–57.9% survival rates against *E. coli* (10^6^), conversely at 0.01–1 mg/mL [41]. MgO NPs at 100 mg/mL were bactericidal against *E. coli* after 1-h incubation despite no antibacterial performance against *S. aureus* within 3-h incubation [42]. In addition, CuO NPs at 25 and 50 μg/mL showed 87% and 92% growth inhibition rates against *E. coli* after 16-h incubation although the growth inhibition rates were not significantly reduced at approximately 2.11% for both concentrations after 1-h incubation [43].

All MO particles themselves had no inactivation activity against phage (Figure 4) because the phage is resistant to environmental stresses such as heat and pH [44]. However, an immunoprotective function of ZnO NPs as virostatic agents has been reported against genital Herpes viruses preventing the viral entry to cells and subsequent infection via generating complexes by charge–charge interaction [45,46]. Cu_2_O NPs also have an in vitro antiviral activity against Hepatitis C inhibiting the viral entry and further infection to cells, with hindrance to viral replication [45,47].

At this point, the hybridization of MO particles with dual UV was tested to investigate the effects of particle size distribution of MO particles and combination with dual UV for enhanced photocatalytic antimicrobial performance against bacteria as well as viruses. The antimicrobial activity of MO particles at multiscale size levels and dual UV of UV-A and UV-C irradiating differential UV-C intensity depending on irradiation time, were analyzed against *E. coli* and phage. In MO particles, ZnO-(1) and CuO particles had multiscale particle size distributions although ZnO-(2) and MgO-(1) particles showed nanoscale size levels, and MgO-(2) and Cu_2_O particles displayed microscale size levels. Multiscale ZnO and CuO particles showed superior antimicrobial activity against *E. coli* (Figure 5) and phage (Figure 6) compared with other scale particles, due to large surface area and multiple scattering under dual UV irradiation [5,6,30]. The large surface area of multiscale ZnO (ZnO-(1) > ZnO-(2) by 4.0-fold) and CuO (CuO > Cu_2_O by 5.6-fold) particles induced charge–charge interaction to cause membrane damage of pathogenic microorganisms for enhanced antimicrobial performance [48,49]. The microstructured particle aggregates with polydispersity also showed multiple scattering phenomena improving particular interactions via mass transfer and exchange in multilevel porosity, including the biomodality of macropores and mesopores for enhanced photocatalysis promoting antimicrobial action [50,51]. Enhanced photocatalytic antimicrobial actions of multiscale MO particles could be explained by multiple scattering of nano- or microstructured MO particle clusters in a hydrodynamic environment connecting to dual UV irradiation. These results were also superior to previously reported data for MO NPs and dual UV hybrids [11].

**Figure 7:**
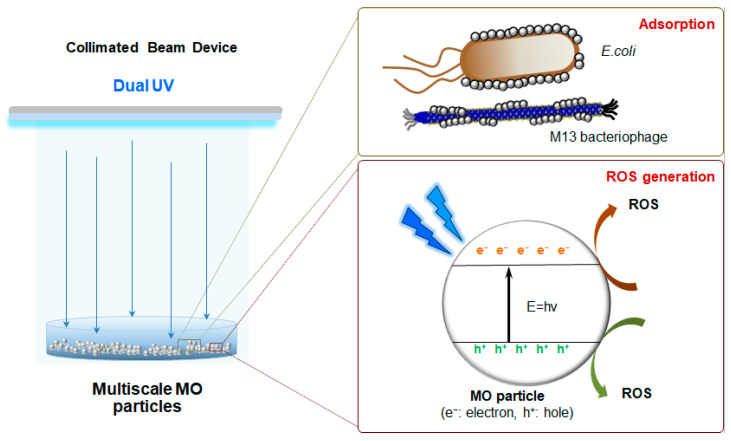
Schematic diagram of main antimicrobial mechanisms of a dual ultraviolet (UV)-multiscale metal oxide (MO) particle system for enhanced antimicrobial action. In water, multiscale MO particles were adsorptive to *E. coli* and phage resulting in biomembrane rupture and cell death. They generated reactive oxygen species (ROS) under dual UV irradiation promoting an electron from valence band to conduction band by a band gap energy.

The antimicrobial mechanisms of multiscale MO particles can be explained by the release of metal ions, particle shape dependency, particle adsorption to the biomembrane of microorganisms, and ROS generation under dual UV irradiation [2,4]. High metal ion release, high aspect ratio in morphology, large surface area, and enhanced photocatalytic activities are critical characteristics of multiscale MO particles for enhanced antimicrobial performance. Specifically, biomembrane adsorption and ROS generation are primarily considered as expected antimicrobial mechanisms that cause biomembrane damage and DNA breaks against microorganisms (Figure 7). The adsorption potential was induced by the physicochemical characteristics of the MO particles, including hydrophobicity, porosity, and dispersibility in an aqueous environment. In ROS generation, multiscale MO particles generate particle aggregates mimicking nano-to-microscale architecture for enhanced photocatalytic performance even at lower concentrations via multiple scattering [52]. In addition, emulating surface defects of multiscale MO particles could improve the antimicrobial activity in a hydrodynamic environment due to their enhanced ROS production from oxygen vacancies [53,54,55]. In particular, the structures of MO NPs or MPs affected the surface oxygen vacancies, resulting in enhanced ROS generation for antimicrobial performance based on electron-hole pair generation [55,56,57]. However, in the case of CuO and Cu_2_O particles, CuO particles reduce the superoxide radicals to produce Cu^+^, and Cu_2_O particles do not generate any superoxide radicals for sustained oxidative stress during redox cycling [58]. In these phenomena, although CuO-Cu_2_O particles generated no superoxide species, superoxide anion radical (O_2_·^−^)) or hydroxyl radical (OH·) was produced through the Fenton reaction. Chelation complex formation or metabolic enzyme damage was caused in microorganisms by ROS from Cu_2_O and CuO particles, respectively. Therefore, multiscale MO particles can be used as alternatives to antibiotic agents for enhanced performance based on differential antimicrobial pathways to overcome drug resistance.

## 5. Conclusions

Multiscale MO particles had nano- to microscale sizes as mixtures of NPs and MPs, which were identified as pure materials, confirmed by atomic compositions and crystallinity, and further evaluated by surface characteristics. In ZnO, MgO, Cu_2_O, and CuO particles, multiscale ZnO and CuO particles showed enhanced antimicrobial activity against *E. coli* as well as phage under dual UV irradiation, based on their physicochemical characteristics. In particular, surface adsorption to biomembrane and ROS generation under dual UV irradiation can be the main mechanisms of antimicrobial action due to large surface area with enhanced mass transfer via multiple scattering. From the results, multiscale MO particles will be promising antibiotic agents to apply in environment, industry, and clinics for near future. It can also be extended to customized multiple combination mixtures of multiscale MO particles as photocatalysts in dual UV hybrid systems for enhanced performance overcoming antibiotic resistance.

## Figures and Tables

**Table 1:** Atomic compositions of metal oxide (MO) particles.

MO Particles	Zn	Al	Mg	Cu	O
ZnO-(1)	78.5 ± 1.6	0.01 ± 0.02	-	-	21.4 ± 1.6
ZnO-(2)	81.9 ± 0.6	-	-	-	18.1 ± 0.6
MgO-(1)	-	-	61.1 ± 1.4	-	38.9 ± 1.4
MgO-(2)	-	-	63.5 ± 2.1	-	36.5 ± 2.1
Cu_2_O	-	-	-	88.7 ± 1.2	11.3 ± 1.2
CuO	-	-	-	75.9 ± 0.3	24.1 ± 0.3

**Table 2:** Surface characteristics of metal oxide (MO) particles.

MO Particles	Surface Area (m^2^/g)	Pore Volume (cc/g)	Pore Size (nm)
ZnO-(1)	46.34	0.1105	9.540
ZnO-(2)	11.58	0.08644	29.86
			
MgO-(1)	25.20	0.2883	45.76
MgO-(2)	0.3997 0.1090 ^1^	0.003593 -	35.96 -
Cu_2_O	0.6036	0.003893	25.80
CuO	3.392	0.01783	21.02

^1^ Krypton.

## Data Availability

The data presented in this study are available in article or supplementary material.

## Supplementary Materials

The following are available online at https://www.mdpi.com/1999-4923/13/2/222/s1, Figure S1: Size distributions of MO particles in the hydrodynamic environment, Figure S2: EDS spectra of MO particles, Figure S3: XPS spectrum of multiscale ZnO-(1) particles, Table S1: Particle size distributions of MO particles in the hydrodynamic environment.
