# Supplementary material for: Multiscale Metal Oxide Particles to Enhance Photocatalytic Antimicrobial Activity against Escherichia coli and M13 Bacteriophage under Dual Ultraviolet Irradiation
Source: Pharmaceutics. 2021 Feb 6;13(2):222. doi: 10.3390/pharmaceutics13020222 (PMC7914579; doi:10.3390/pharmaceutics13020222)
Supplement: Supplementary file 1 [file pharmaceutics-13-00222-s001.pdf]

pharmaceutics

MDPI

Supplementary Materials: Multiscale Metal Oxide Particles to Enhance Photocatalytic Antimicrobial Activity Against Escherichia coli and M13 Bacteriophage under Dual Ultraviolet Irradiation

Su-Eon Jina* and Hyo-Eon Jinb*

1. Size measurement

Size was measured using light scattering (ELS-Z, OTSUKA Electronics Co., Ltd., Osaka, Japan) with a standard cell. Metal oxide (MO) particles were dispersed in 30% ethanol-distilled water. For the measurements, particles were diluted with absolute ethanol prior to the analysis to obtain the appropriate intensity. The mean diameters (nm) of particles were determined at room temperature.

Publisher's Note: MDPI stays neutral with regard to jurisdictional claims in published maps and institutional affiliations.

Copyright: © 2020 by the authors. Submitted for possible open access publication under the terms and conditions of the Creative Commons Attribution (CC BY) license (http://creativecommons.org/licenses/by/4.0/).

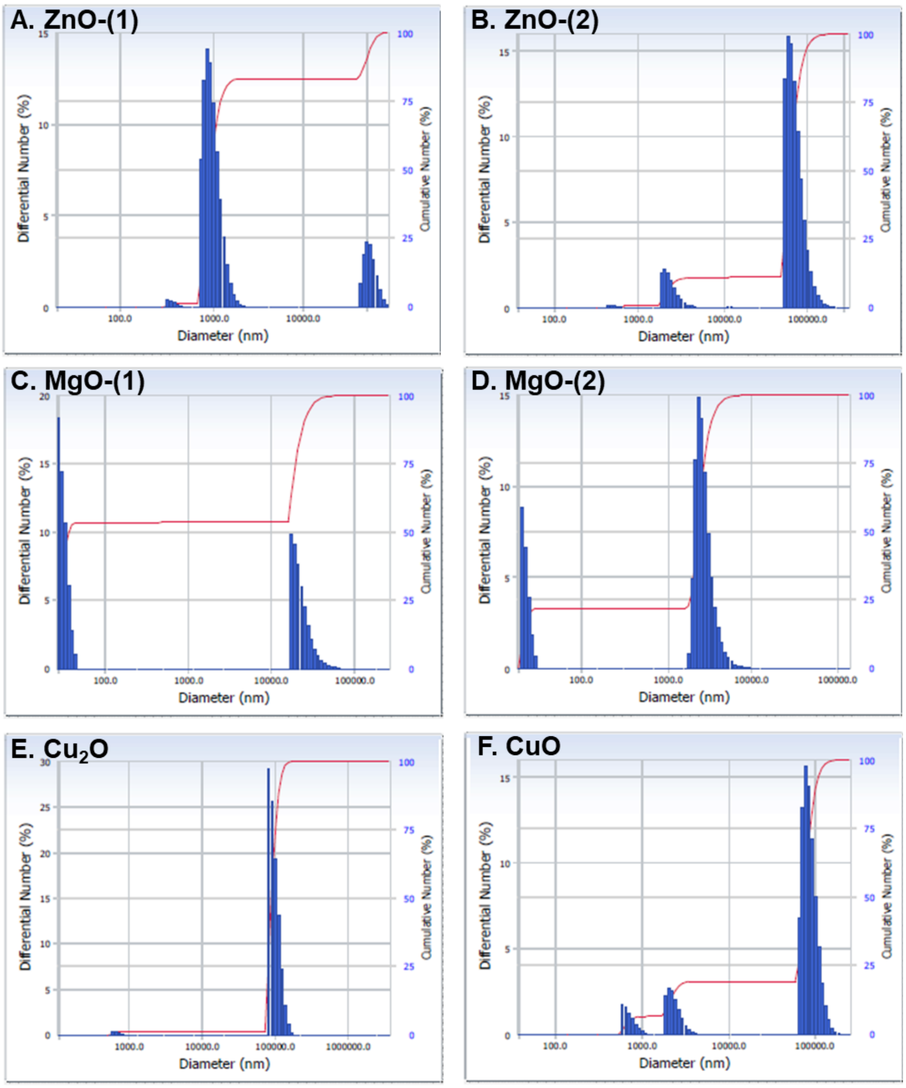

Figure S1. Size distributions of MO particles in the hydrodynamic environment: (A) ZnO-(1), (B) ZnO-(2), (C) MgO-(1), (D) MgO-(2), (E) Cu_2O, and (F) CuO. They were monitored using the number-weighted mode. Flocculates or aggregates of particles occurred up to 100 μm-scale.

Table S1. Particle size distributions of MO particles in the hydrodynamic environment.

Particles	Particle size distribution (Number)			
	1st peak	2nd peak	3rd peak	4th peak
ZnO-(1)	367 ± 38	1038 ± 225†	58101 ± 9041	-
ZnO-(2)	530 ± 72	2457 ± 516	13058 ± 1424†	73167 ± 18421
MgO-(1)	34 ± 4†	653 ± 139	23719 ± 6656	-
MgO-(2)	24 ± 3	430 ± 98†	2849 ± 781	-
Cu_2O	665 ± 62	100177 ± 16963†	-	-
CuO	749 ± 121	2495 ± 450	93576 ± 19186†	-

† The largest peak in the particle size distirbution plot.

2.Energy-dispersive X-ray spectroscopy (EDS)

EDS analysis, equipped with a FE-SEM (JSM-7100F, Jeol Ltd., Tokyo, Japan) operated at an acceleration voltage of 15.0 kV, was performed at three points on the surface of MO particles to quantitatively determine the compositions of elements.

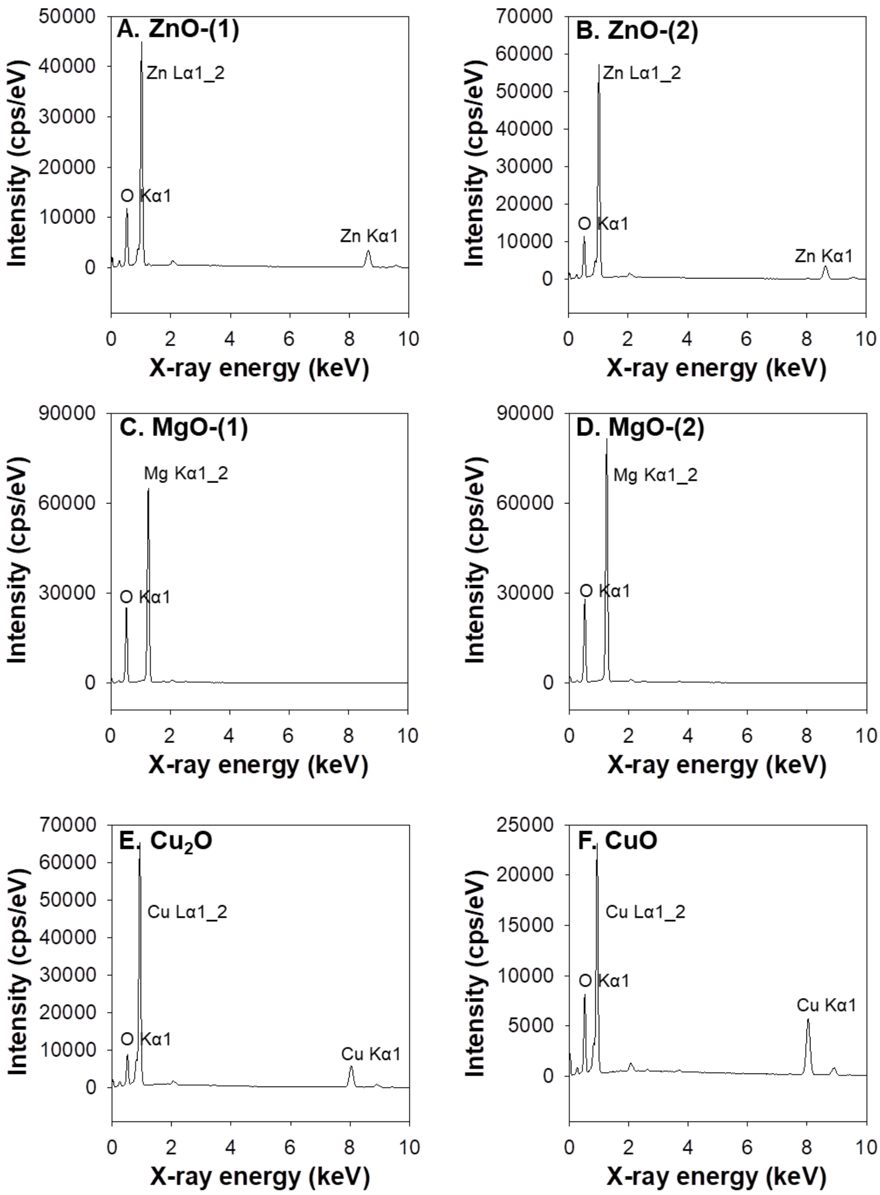

Figure S2. EDS spectra of MO particles: (A) ZnO-(1), (B) ZnO-(2), (C) MgO-(1), (D) MgO-(2), (E) Cu_2O, and (F) CuO.

3.X-ray photoelectron spectroscopy (XPS)

The atomic compositions of inner surface of the tubes were determined using XPS (K-Alpha, Thermo Scientific, Thermo Fisher Scientific Inc., Hillsboro, OR, USA) with a source of monochromated Al K-alpha. A flood gun was used for charge compensation.

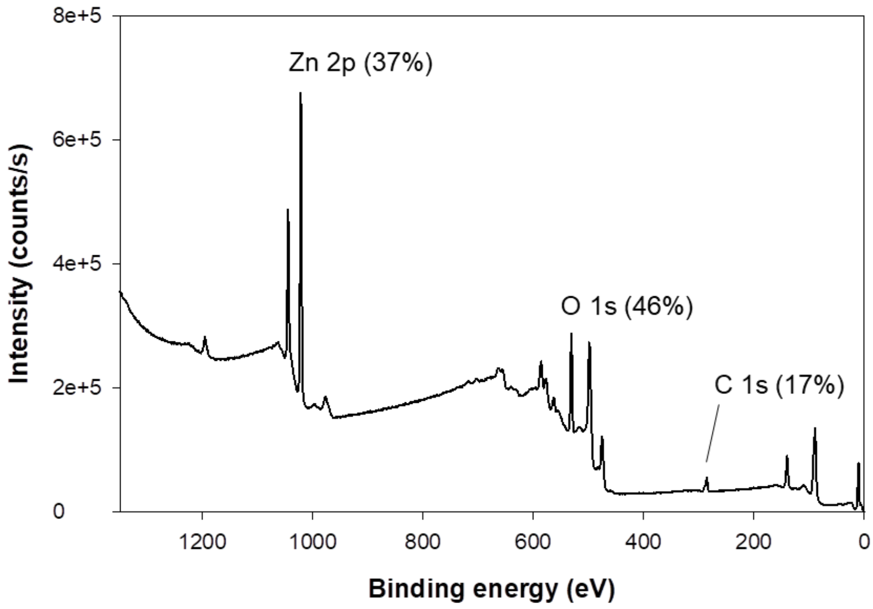

Figure S3. XPS spectrum of multiscale ZnO-(1) particles.
